# A Novel Sulfonamide, Molecularly Imprinted, Upconversion Fluorescence Probe Prepared by Pickering Emulsion Polymerization and Its Adsorption and Optical Sensing Performance

**DOI:** 10.3390/molecules28083391

**Published:** 2023-04-12

**Authors:** Qidi Pan, Zhe Gao, He Meng, Xianghua Guo, Meitian Zhang, Yiwei Tang

**Affiliations:** 1 College of Food Science and Technology, Hebei Agricultural University, Baoding 071001, China; 2 Qian’an Agricultural and Rural Bureau, Qian’an 064400, China

**Keywords:** molecularly imprinted polymer, Pickering emulsion polymerization, upconversion fluorescence probe, sulfonamides

## Abstract

A novel, molecularly imprinted, upconversion fluorescence probe (UCNP@MIFP) for sulfonamide sensing was fabricated by Pickering emulsion polymerization using UCNP@SiO_2_ particles as the stabilizer and sulfamethazine/sulfamerazine as the co-templates. The synthesis conditions of the UCNP@MIFP were optimized, and the synthesized probe was characterized by scanning electron microscopy, Fourier transform infrared spectrometer, thermogravimetric analyzer, and fluorescence spectrometer. The UCNP@MIFPs showed a good adsorption capacity and a fast kinetic feature for the template. The selectivity experiment revealed that the UCNP@MIFP has a broad-spectrum molecular recognition capability. Good linear relationships were obtained over the concentration range of 1–10 ng/mL for sulfamerazine, sulfamethazine, sulfathiazole, and sulfafurazole, with low limits of detection in the range of 1.37–2.35 ng/mL. The prepared UCNP@MIFP has the potential to detect four sulfonamide residues in food and environmental water.

## 1. Introduction

Sulfonamides are a class of bacteriostatic drugs that inhibit bacterial growth and reproduction by interrupting the synthesis of bacterial folate and nucleotides [1,2]. Due to their broad antibacterial spectrum, easy production, low price, stable properties, and convenient taking, they have been widely used in human and veterinary medicines for the prevention and treatment of diseases caused by bacteria [3]. However, serious environmental problems and food pollution from the overdose and extensive use of sulfonamides have also received considerable attention [4]. Therefore, it is necessary to obtain an accurate and rapid measurement method to sense sulfonamides.

To date, a large number of analytical methods, including high-performance liquid chromatography [5], high-performance liquid chromatography-tandem mass spectrometry [6], capillary electrophoresis [1], enzyme-linked immunosorbent assay [7], and surface-enhanced Raman spectroscopy [8], have been developed for sulfonamide determination. However, these methods have some disadvantages, such as complex pretreatment procedures, poor portability, the requirement of expensive instruments, low sensitivity, and/or poor specificity.

Fluorescence probes have attracted considerable attention due to their excellent sensitivity, fast response, and stable emission [9]. Upconversion nanoparticles (UCNPs) are a unique class of lanthanide-ion-doped optical nanomaterials featuring a wealth of electronic transitions within 4f electron shells [10]. Compared with other fluorescent materials (i.e., quantum dots and organic dyes), UCNPs can upconvert two or more lower-energy photons into one high-energy photon with high quantum yield, large anti-Stokes’s shift, excellent photostability, and low autofluorescence background, which are favorable for applications in the detection field [11]. UCNP-based fluorescence probes using antibodies and aptamers as molecular recognition elements have been fabricated to sense mycotoxin [12], pathogenic bacteria [13], veterinary drugs [14], and pesticides [15]. In addition to biological receptors (i.e., antibodies and aptamers), molecularly imprinted artificial materials as receptors for molecules of interest have received considerable attention due to their excellent advantages, such as low cost, easy production, high selectivity, and stable physicochemical properties [16].

Generally, molecularly imprinted fluorescence probes (MIFPs) are constructed by bulk imprinting [17], surface imprinting [18,19], and the sol-gel imprinting method [15]. Of these, surface imprinting is the most preferred method to prepare MIFPs that have outstanding access to target molecules [20]. Pickering-emulsion-polymerization-based surface imprinting has become a promising alternative to synthesize the desired MIFPs due to its simplicity, high yield of polymers, and the lower usage of hazardous surfactants that have environmental consequences [21]. Pickering emulsions stabilized by hemoglobin, CdTe quantum dots, and silica nanoparticles have been used to prepare MIFPs for hemoglobin, *Listeria monocytogenes*, and bisphenol A detection [22,23,24], but little work has been reported on the synthesis of MIFPs via Pickering emulsion polymerization using modified UCNP nanoparticles as stabilizers.

To achieve the purpose of the adsorption of multiple substances at one time, dual/multi-template molecularly imprinted polymers (MIPs) have been prepared [25]. Caffeine-H_3_PO_4_ and *N*-(diethoxyphosphinothioyl-l-phenylalanine) were employed as dual dummy templates to prepare the MIPs that can adsorb seven organophosphorus pesticides [26]. Singh et al. prepared a MIP sensor for the simultaneous determination of 4-nitrophenol and hydroquinone using dual template imprinting technology [27]. In this study, we first synthesize a UCNP-based MIFP (UCNP@MIFP) by Pickering emulsion polymerization using silica-coated upconversion nanoparticles (UCNPs@SiO_2_) as the stabilizer and sulfamerazine/sulfamethazine as the co-templates. The prepared UCNP@MIFP can selectively capture multiple sulfonamides (i.e., sulfamerazine, sulfamethazine, sulfathiazole, and sulfafurazole), resulting in a fluorescence quenching. Good relationships between sulfonamide concentrations and the fluorescence intensities of the UCNP@MIFP were obtained.

## 2. Results and Discussion

### 2.1. Synthesis of the UCNP@MIFPs

**Scheme 1:**
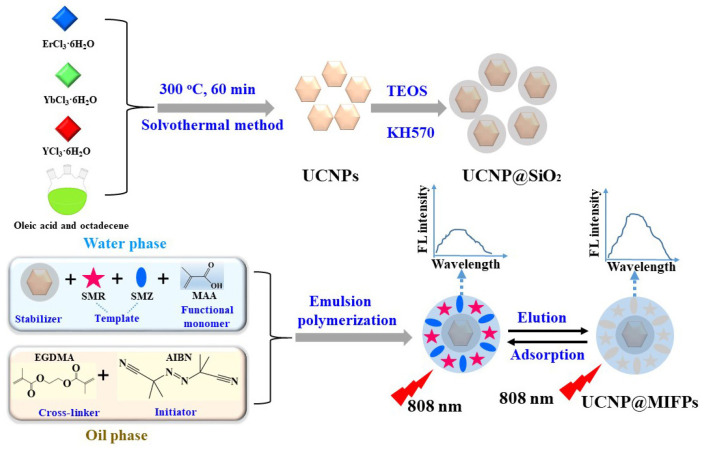
Scheme of the fabrication of the multifunctional UCNP@MIFPs.

As shown in Scheme 1, UCNPs were synthesized and modified with silica to introduce abundant surface silanol groups, carbonyl groups, and carbon–carbon double bonds, making them suitable as a Pickering-type stabilizer [28]. Then, the UCNP@MIFPs were fabricated by Pickering emulsion imprinting polymerization using SMZ/SMR as the co-templates, MAA as the functional monomer, EGDMA as the cross linker, and UCNP@SiO_2_ as the stabilizer.

The molar ratio between template, functional monomer, and cross linker is a crucial factor affecting the target-binding properties of the UCNP@MIFPs. The UCNP@MIFPs were prepared using various template/functional monomer/cross linker molar ratios (1:1:4, 1:1:6, 1:1:8, 1:2:4, 1:2:6, 1:2:8, 1:2:8, 1:4:4, 1:4:6, and 1:4:8). The adsorption capacities of UCNP@MIFPs and the corresponding UCNP@NIFPs (the non-imprinted fluorescence probe based on UCNP) for SMZ are listed in Table 1. These results indicate that the template/functional monomer/cross linker molar ratio of 1:4:4 was the best to synthesize the probe with the highest imprinting factor of 3.67 (imprinting factor = *Q*_UCNP@MIFP_/*Q*_UCNP@NIFP_), showing the recognition sites formed on the surface of the UCNP@MIFP. Therefore, the molar ratio of 1:4:4 between the template, functional monomer, and cross linker was chosen to prepare the UCNP@MIFPs.

### 2.2. Characterization

**Figure 1:**
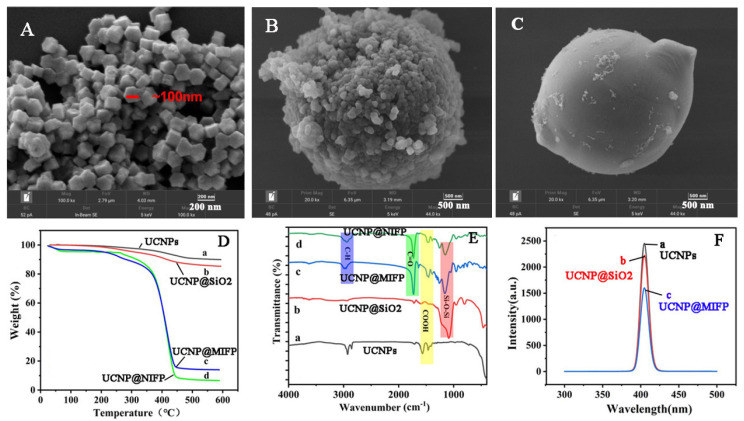
SEM image of UCNPs (**A**), UCNP@MIFP (**B**), UCNP@NIFP (**C**), TGA (**D**), FT−IR (**E**), and fluorescence spectra (**F**).

The morphologies of the prepared materials were characterized by scanning electron microscopy. As illustrated in Figure 1A, UCNPs have a hexahedral-like morphology, with a mean size of ~100 nm. The synthesized UCNP@MIFP is spherical with a rough surface (Figure 1B), and the UCNP@NIFP presents a smooth surface (Figure 1C). The thermogravimetric analysis results (Figure 1D) show that 85% weight loss for UCNP@MIFP was found, resulting from the thermal degradation of the MIP layer. To further ascertain the imprinting process, the FT-IR spectra of the synthesized materials were obtained (Figure 1E). The bands at 2927 cm^−1^ and 2855 cm^−1^ in the spectrum of the hydrophobic UCNPs (black line in Figure 1E) were attributed to the –CH_2_- of oleic acid, and the peaks of 1421 cm^−1^ and 1560 cm^−1^ were attributed to the vibrational mode of –COOH [29]. The Si-O-Si band at 1090 cm^−1^ was identified in the UCNP@SiO_2_ (red line in Figure 1E), indicating the SiO_2_ shell was successfully obtained. The new bands at 1731 cm^−1^ and 1161 cm^−1^ were assigned the stretching vibration of C=O and C−O groups of EGDMA, which was used for the preparation of UCNP@MIFP and UCNP@NIFP. The fluorescence spectra of the UCNPs, UCNP@SiO_2_, and UCNP@MIFP (Figure 1F) show that the silica coating and MIP shell resulted in a reduced fluorescence signal. These results confirm the successful fabrication of the upconversion fluorescence probe.

### 2.3. Static Adsorption

**Figure 2:**
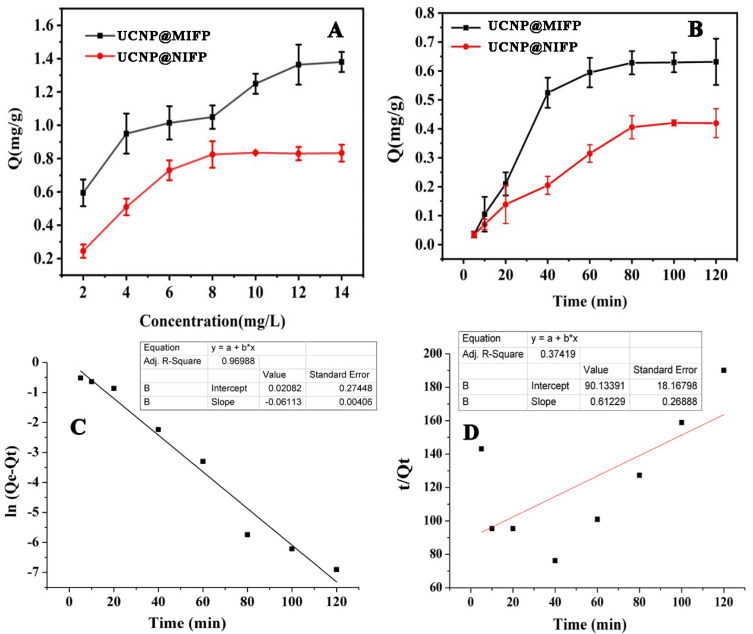
Static adsorption capacity of UCNP@MIFP and UCNP@NIFP (**A**). Uptake kinetic plot of SMZ onto the UCNP@MIFP and UCNP@NIFP (**B**) and the pseudo−first−order (**C**) and pseudo−second−order (**D**) models for the adsorption of SMZ onto the UCNP@MIFP.

To study the adsorption capacity of the prepared UCNP@MIFP and UCNP@NIFP, a static adsorption test was carried out. As shown in Figure 2A, the adsorption capacities of UCNP@MIFP and UCNP@NIFP increase with increasing initial concentrations, and the greater static adsorption capacity of UCNP@MIFP for SMZ was observed at the same concentration. When the concentration is 12 mg/L, the amount of SMZ adsorbed to the UCNP@MIFP (1.38 mg/g) is more than 1.66 times than that of UCNP@NIFP (0.83 mg/g). The increased adsorption capacity is mainly owed to the specific affinity binding sites in the UCNP@MIFP.

The static adsorption of the UCNP@MIFP was analyzed by Freundlich and Langmuir models expressed by the following equations [19]:

(1) $$\ln Q=n\;\ln C_{e}+\ln k$$

(2) CeQ=1Qmk1+CeQm

where *Q* is the adsorption capacity of the UCNP@MIFP at equilibrium, *Q_m_* is the maximum adsorption capacity of the UCNP@MIFP, *C_e_* is the concentration of the target at equilibrium, *k*_1_ is the adsorption equilibrium constant in the Langmuir model, and *k* and *n* are the adsorption constant and heterogeneity factor in the Freundlich model, respectively. As shown in Figures S1 and S2 (Supplementary Materials), the Langmuir model can fit the experimental data better than the Freundlich one, based on the correlation coefficient (*R*^2^) value of both models.

### 2.4. Adsorption Kinetics

Dynamic adsorption was performed to investigate the binding rate of the UCNP@MIFP to the target SMZ. The adsorption capacity of the prepared upconversion fluorescence probe at different incubation times is shown in Figure 2B. A rapid adsorption rate was found at the beginning and reached the adsorption equilibrium within 80 min.

To better understand the adsorption processes of the target SMZ onto the UCNP@MIFP, the adsorption data were fitted by using a pseudo-first-order kinetic model (Equation (3)) and a pseudo-second-order kinetic model (Equation (4)):

(3) $$\ln\left(Q_{e}-Q_{t}\right)=\ln Q_{e}-k_{1}t$$

(4) tQt=1k2Qe2+tQe

where *Q_e_* and *Q_t_* are the adsorption capacities of the UCNP@MIFP at equilibrium and at time *t*, respectively, and *k*_1_ and *k*_2_ are the adsorption rate constants of the pseudo-first-order and pseudo-second-order kinetic models, respectively. As shown in Figure 2C,D, the correlation coefficient of the pseudo-first-order kinetic model (*R*^2^ = 0.97) was better than that of the pseudo-second-order kinetic model (*R*^2^ = 0.37). Therefore, the adsorption kinetics of the UCNP@MIFP probe to the target molecule can be explained well by the first-pseudo kinetic arrangement, suggesting the physical process might be considered as the rate-determining step during the entire rebinding process [30].

### 2.5. Incubation Time

**Figure 3:**
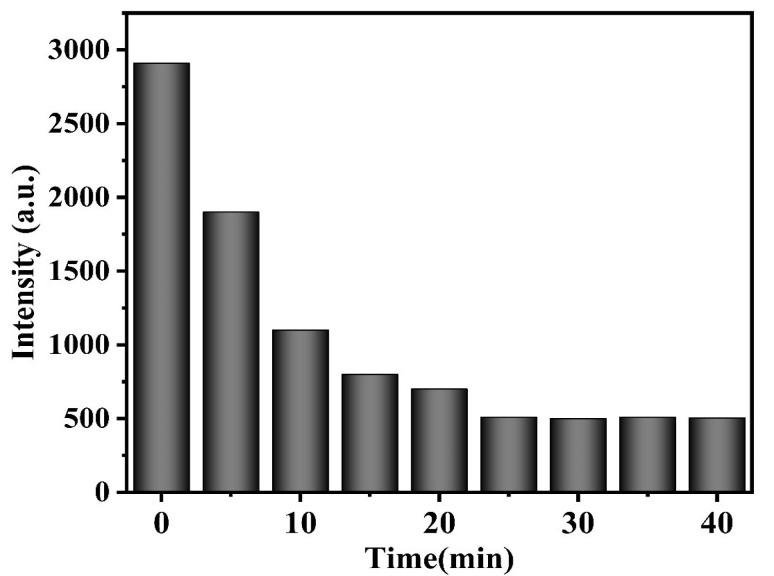
Fluorescent intensity of the UCNP@MIFP incubated with the target at different time.

The incubation time between the UCNP@MIFP probe and the target was determined by recording the fluorescent intensity at different time points. As shown in Figure 3, the fluorescence intensity decreased rapidly after adding the SMZ target molecules. The stable fluorescent intensity was identified after 30 min and then leveled off, which can be attributed to the reaching of equilibrium [31]. We thus adopted the incubation time of 30 min between the UCNP@MIFP and the SMZ target for further experiments.

### 2.6. Possible Fluorescence Quenching Mechanism

**Figure 4:**
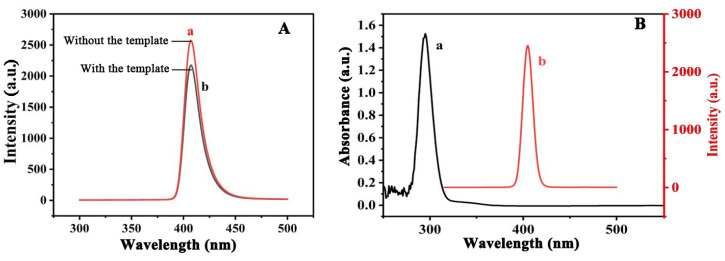
(**A**) Fluorescence spectra of UCNP@MIFP without (a) and with the template (b), (**B**) UV-vis absorption spectrum of SMZ (a) and the fluorescence spectrum of UCNP@MIFP (b).

The fluorescence spectra of UCNP@MIFP before and after removal of the SMZ template molecule are shown in Figure 4A. A significant fluorescence enhancement of the UCNP@MIFP was found after removing the template, indicating an interaction between the fluorescent probe and the template molecules. Because of the lack of overlap between the fluorescent spectrum of the UCNP@MIFP and the UV-vis absorption spectrum of the target (Figure 4B), fluorescence resonance energy transfer as a possible fluorescence quenching mechanism could be ruled out. A photoinduced charge transfer facilitated by hydrogen bonding is another important fluorescence quenching mechanism [19,32,33]. Moreover, Wu et al. found tyrosine can quench the fluorescence of UCNPs by photoinduced electron transfer [34]. Therefore, we speculated that, when the target molecules (e.g., SMZ and SMR) was captured by the probe via the hydrogen bond, the emitted fluorescence from the UCNP@MIFP may cause charge transfer from the lone pair of electrons to the hydrogen bond, resulting in photoinduced electron transfer quenching.

### 2.7. Selectivity of the UCNP@MIFP

**Figure 5:**
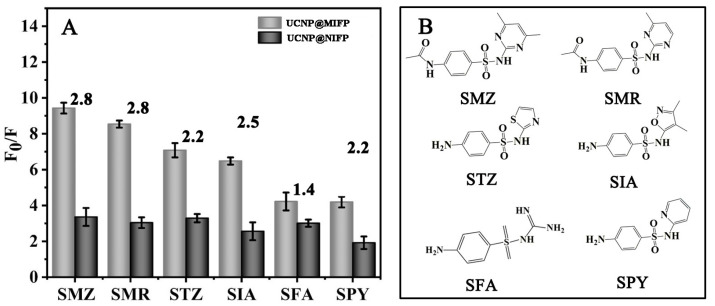
The *F*_0_/*F* and selective coefficient (selective coefficient = *F*_0_/*F*_UCNP@MIFP_/*F*_0_/*F*_UCNP@NIFP_) of the six sulfonamides (**A**) and the chemical structures of the six sulfonamides (**B**).

The capability of multiplex analysis of the probe was assessed by employing six sulfonamides (i.e., SMR, SMZ, STZ, SIA, SFA, and SPY) to quench the UCNP@MIFP and UCNP@NIFP at the same concentration. The F_0_/F and selective coefficient (selective coefficient = *F*_0_/*F*_UCNP@MIFP_/*F*_0_/*F*_UCNP@NIFP_) of six sulfonamides are shown in Figure 5, indicating that four sulfonamides (SMR, SMZ, STZ, and SIA) significantly quenched the fluorescence of the UCNP@MIFP, but other two sulfonamides (SFA and SPY) had negligible influence on the fluorescence quenching. Moreover, these six sulfonamides had an almost similar influence on the fluorescent intensity of the UCNP@NIFP, suggesting that no specific binding sites were available in the non-imprinted polymers. Therefore, UCNP@MIFP probe can be used for the multiplex analysis of sulfonamides, with a good selective recognition ability.

### 2.8. Fluorometric Analysis

**Figure 6:**
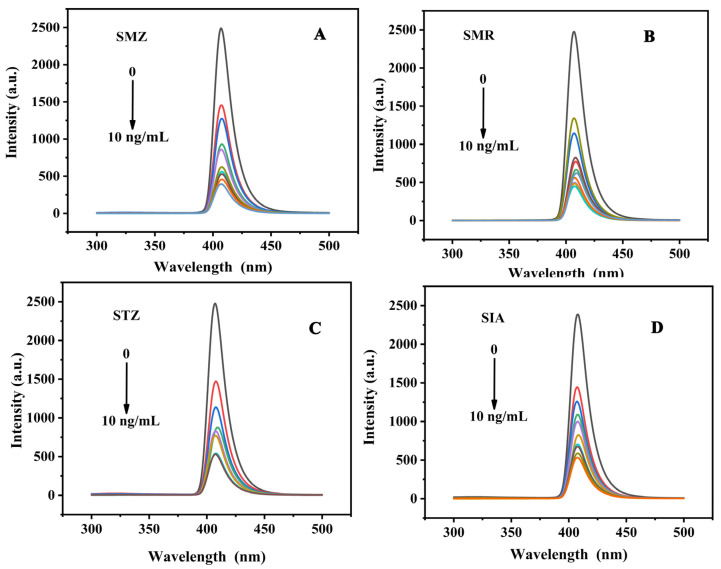
The fluorescence spectra of the UCNP@MIFP incubated with different concentrations of SMZ (**A**), SMR (**B**), STZ (**C**), and SIA (**D**).

Under the optimized sensing conditions, the fluorescence spectra of UCNP@MIFP probe before and after capturing the target are shown in Figure 6, indicating that the fluorescence intensity decreased drastically with increasing SMR, SMZ, STZ, and SIA concentrations.

In this study, the fluorescence quenching was analyzed by the Stern–Volmer equation, *F*_0_/*F* = 1 + *K*_sv_*C*_q_ (*F*_0_ is the initial fluorescence intensity in the absence of the quencher, *F* is the fluorescence intensity in the presence of the analyte, *K*_sv_ is the quenching constant of the quencher, and *C*_q_ is the concentration of the analyte). Good relationships between sulfonamide (SMR, SMZ, STZ, and SIA) concentrations and the fluorescence intensities of the UCNP@MIFP were observed in the range from 0 to 10 ng/mL. The linear regression equation and limit of detection (LOD, 3S/N) are listed in Table 2, indicating that the prepared UCNP@MIFP fluorescence probe has the potential to detect four sulfonamide residues in food.

Compared with the electrochemical sensor method [35], lanthanide metal–organic-framework-based paper strip sensor [36], and carbon-dot-embedded photonic-crystal molecularly imprinted sensor array [2], the proposed probe is more sensitive. Moreover, the upconversion-nanoparticle-based fluorescence probe has no autofluorescence signal contribution from the sample matrix due to the use of infrared light as the excitation light source, which has the more attractive advantages of low toxicity, large anti-Stokes shift, fascinating photostability, robust penetration ability of biological tissue, and minimum autofluorescence interference [37].

### 2.9. Stability of the UCNP@MIFP

**Figure 7:**
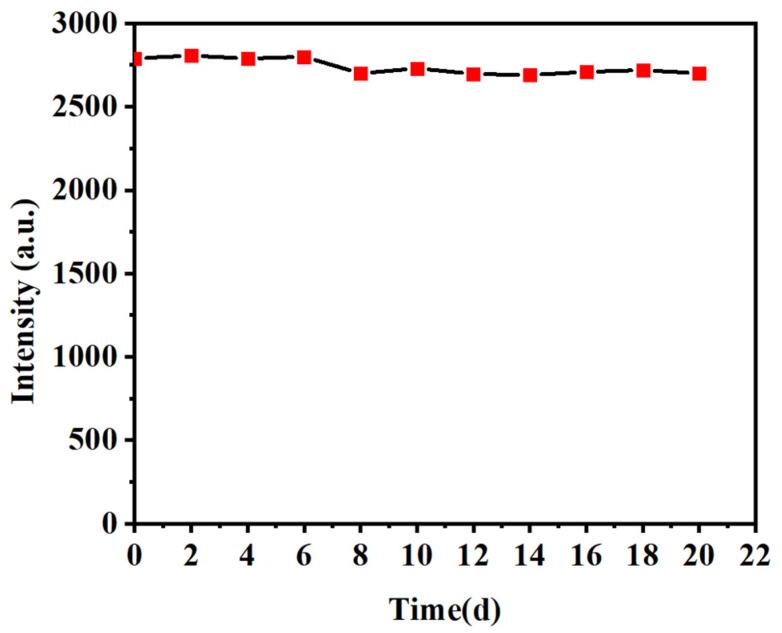
Fluorescence intensities of the UCNP@MIFP during different storage times.

The stability of the prepared UCNP@MIFP was evaluated, and the fluorescence intensity of the probe was repeatedly recorded. As shown in Figure 7, the corresponding fluorescence intensity of the UCNP@MIFP remains unaffected within 20 days with no significant decrease, suggesting that the probe owns good photobleaching resistance and fluorescence stability.

### 2.10. Analysis of Real Samples

To evaluate the application of the UNCP@MIFP-based fluorescent analysis method, a spike-and-recovery test was carried out by introducing three different concentrations into water and fish muscle samples. As listed in Table 3, the average recoveries of the analyzed samples were in the range of 96.00–100.33%, and the relative standard deviations (RSDs) were lower than 3.5%. The accuracy of the UNCP@MIFP-based fluorescent analysis method was compared with the determination of the spiked samples using the HPLC method. The results are shown in Table 3, which shows no significant difference between the two methods.

## 3. Materials and Methods

### 3.1. Chemicals and Material

Erbium (III) chloride hexahydrate (ErCl_3_·6H_2_O), yttrium (III) chloride hexahydrate (YCl_3_·6H_2_O), ytterbium (III) chloride hexahydrate (YbCl_3_·6H_2_O), oleic acid (OA), octadecene (ODF) NaOH, NH_4_F, cyclohexane, methacrylic acid (MAA), ethylene glycol dimethacrylate (EGDMA), and azobisisobutyronitrile (AIBN) were purchased from Bailingwei Technology Co., Ltd. (Beijing, China). Sulfamerazine (SMR), sulfamethazine (SMZ), sulfathiazole (STZ), sulfafurazole (SIA), sulfacetamide (SFA), and sulfapyridine (SPY) were purchased from Aladdin Biochemical Technology Co., Ltd. (Shanghai, China). Methanol and formic acid were of chromatographic grade and purchased from Thermo Fisher Scientific (China) Co., Ltd. (Shanghai, China). Other chemicals were obtained from Tianjin Chemical Reagent Factory (Tianjin, China).

### 3.2. Apparatus

The FT-IR spectra of the prepared particles were recorded in KBr using a Nicolet Nexus 470 FT-IR spectrophotometer (Thermo Electron, Waltham, MA, USA) at room temperature. A JEOL JEM 2100 transmission electron microscope (JEOL, Tokyo, Japan) coupled with energy dispersive X-ray was employed to characterize the morphology, size, and elemental composition. The thermogravimetric analysis of the samples was carried out using a TG/DSC Star system thermogravimetric analyzer (Methler Toledo, Zurich, Switzerland). The Upconversion fluorescence spectra were recorded on an F-320 fluorescence spectrophotometer (Gangdong, Tianjin, China) at the excitation wavelength of 808 nm. A UV-Vis spectrophotometer (UV-3150, GL Sciences, Tokyo, Japan) was used to assess the adsorption properties of the fluorescence probe.

### 3.3. Synthesis of the Upconversion Nanoparticles (UCNPs)

Briefly, YCl_3_·6H_2_O (242.7 mg), YbCl_3_·6H_2_O (69.8 mg), ErCl_3_·6H_2_O (7.7 mg), 3 mL oleic acid (OA), and 17 mL octadecene (ODF) were added into a three-necked flask. Then, the mixture was heated at 160 °C under nitrogen protection with constant stirring for 30 min. After cooling to room temperature, NaOH (100 mg), NH_4_F (148 mg), and methanol (10 mL) were slowly added to the flask under stirring. Thirty minutes later, the mixture was heated at 85 °C to remove the methanol, and then heated to 300 °C for 60 min. After the flask cooled to room temperature, the UNCPs were obtained by centrifugation and washed with ethanol and the mixed ethanol/cyclohexane solvent (1:1; *v*/*v*), and dried in an oven at 60 °C for 12 h.

### 3.4. Fabrication of Silica-Coated Upconversion Nanoparticles (UCNP@SiO_2_)

The UCNPs (100 mg) were dispersed in cyclohexane (10 mL) containing IGEPAL CO-520 (5 mL) using an ultrasonic oscillator. After 30 min, NH_3_·H_2_O (0.80 mL) was injected into the mixture with constant stirring. Tetraacetoxysilane (0.20 mL) and KH570 (4.0 mL) were then added and the mixture was stirred for 12 h. The UCNP@SiO_2_ was obtained via centrifugation and washed with ethanol three times, and dried at 60 °C for 12 h.

### 3.5. Preparation of the UCNP-Based MIFPs (UCNP@MIFPs)

The UCNP@MIFPs were fabricated via Pickering emulsion polymerization employing UCNP@SiO_2_ nanoparticles as the stabilizer. First, 30 mg of UCNP@SiO_2_ were mixed with 15 mL of ultrapure water containing the co-templates (0.5 mmol SMR and 0.5 mmol SMZ) and functional monomer MAA (4 mmol) under ultrasonic conditions, and the obtained mixture was used as the water phase. The uniform solution of toluene (1 mL), EGDMA (4 mmol), and AIBN (20.0 mg) was obtained by stirring magnetically for 20 min, which was used as the oil phase. Then, the water and oil phases were both moved into a round flask, which was shaken vigorously for 5 min to obtain the Pickering emulsion. After removing the oxygen dissolved in the emulsion by blowing nitrogen for 5 min, the flask was sealed and placed in an oil bath at 65 °C for 24 h. After polymerization, the UCNP@MIFPs were obtained. To extract the template from the fluorescence probe, UCNP@MIFPs were washed with a methanol/acetic acid (9:1, *v*/*v*) solution using the Soxhlet extraction method until no template was detected in the washing solution by a UV-vis spectrometer. The resultant UCNP@MIFPs were dried at 50 °C.

For comparison, the non-imprinted fluorescence probe based on UCNPs (UCNP@NIFP) was prepared in the same conditions without the addition of the template.

### 3.6. Adsorption Kinetic Test

To assess the adsorption kinetics of the SMZ target onto the UCNP@MIFP and UCNP@NIFP, 10 mg of the fluorescence probe and 10 mL of SMZ methanol solutions (6 mg/L) were mixed in 50 mL round flasks. After shaking for 5, 10, 20, 40, 60, 80, 100, and 120 min at room temperature, the mixture was centrifuged at 8000 r/min for 5 min. The SMZ concentration in the supernatant was analyzed using a UV-Vis spectrophotometer at 298 nm.

The adsorption capacity was calculated by Equation (5) according to the difference in SMZ concentration before and after adsorption,

(5) $$Q=\left(C_{0}-C_{a}\right)\times V/m$$

where *Q* (mg/g) is the adsorption capacity, *C*_0_ is the initial concentration of the SMZ solution (mg/L), *C_a_* is the SMZ concentration of the supernatant after adsorption (mg/L), *V* is the volume of the initial SMZ solution (mL), and *m* is the mass of UCNP@MIFP and UCNP@NIFP (g).

### 3.7. Static Adsorption Test

The static adsorption studies were carried out to assess the adsorption capacity of the UCNP@MIFP and UCNP@NIFP. The fluorescence probe (10 mg) was mixed with different initial concentrations of the SMZ methanol solution (2–14 mg/L) with shaking for 80 min. After being centrifuged at 8000 r/min for 5 min, the SMZ concentration in the supernatant was analyzed using a UV-Vis spectrophotometer at 298 nm. The adsorption capacity was determined by Equation (5).

### 3.8. Fluorescence Analysis

The UCNP@MIFP (2 mg) and sulfonamide methanol solution (0.40 mL) were incubated in a slit cuvette for 30 min at room temperature, followed by recording the fluorescence spectrum of each solution in the wavelength range of 300–500 nm upon the excitation of 808 nm. Slit widths (10 nm), scan speed (240 nm/min), quartz cell (1 cm path length), and the excitation voltage (400 V) were kept constantly within each dataset. Each experiment was carried out in triplicate.

### 3.9. Selective Adsorption

To certify the selectivity of UCNP@MIFP, the structural analogs (i.e., STZ, SIA, SFA, SPY, SMZ, and SMR) (Figure 5B for the structures) were prepared at the same concentrations. These solutions were incubated in cuvettes containing 2 mg of UCNP@MIFP or UCNP@NIFP individually, and the changes in fluorescence were monitored.

### 3.10. Sample Analysis

Each water and minced fish muscle sample (1 g) was individually spiked with 1 mL of the SMR, SMZ, STZ, and SIA methanol solutions at three different concentrations (2 μg/L, 6 μg/L, and 8 μg/L). Then, the spiked samples were stirred for 1 min and kept at 4 °C for 8 h. After adding 30 mL of the acetonitrile–formic acid (99:1; *v*/*v*) solvent, the mixture was shaken for 3 min, followed by centrifugation at 4000 rpm for 10 min. The supernatant was collected. Duplicate extraction procedure was carried out, and the supernatant was combined. The solution was evaporated using a nitrogen blowing instrument and a rotary evaporator. The residues were dissolved in 1 mL of methanol for further analysis.

## 4. Conclusions

An efficient and general strategy based on Pickering emulsion polymerization was proposed to construct a UCNP@MIFP using UCNPs@SiO_2_ particles as the stabilizer and sulfamethazine/sulfamerazine as the co-templates. The upconversion fluorescence probe can selectively capture SMR, SMZ, STZ, and SIA molecules with a fast binding rate and a good adsorption capacity. Good linear relationships were obtained between the fluorescence intensity of the UCNP@MIFP and the concentrations of the targets, which has a large application potential in the monitoring and quality control of sulfonamide residues in food and environmental water.

## Figures and Tables

**Table 1:** Adsorption capacities (*Q*) of the probes prepared at different molar ratios between the template, functional monomer, and cross linker and the corresponding imprinting factor (*α*).

Fluorescence Probes	Template:MAA:EGDMA	*Q*_UCNP@MIFP_ ± SD ^a^	*Q*_UCNP@NIFP_ ± SD	*α*
Probe 1	1:1:4	0.22 ± 0.0068	0.10 ± 0.0035	2.20
Probe 2	1:1:6	0.23 ± 0.0055	0.09 ± 0.0021	2.50
Probe 3	1:1:8	0.24 ± 0.0070	0.07 ± 0.0032	3.43
Probe 4	1:2:4	0.10 ± 0.0038	0.04 ± 0.0009	2.50
Probe 5	1:2:6	0.78 ± 0.0120	0.28 ± 0.0108	2.79
Probe 6	1:2:8	0.18 ± 0.0052	0.18 ± 0.0068	1.00
Probe 7	1:4:4	0.33 ± 0.0122	0.09 ± 0.0040	3.67
Probe 8	1:4:6	0.30 ± 0.0091	0.16 ± 0.0005	1.88
Probe 9	1:4:8	0.22 ± 0.0072	0.12 ± 0.0049	1.83

^a^ SD: Standard deviation.

**Table 2:** Stern–Volmer equations with correlation coefficient, linear range, and LOD.

Target	Stern–Volmer Equation	*R* ^2^	Linear Range (ng/mL)	LOD (ng/mL)
SMZ	*F*_0_/*F* = 0.50 *C*_q_ + 1.13	0.98	1–10	1.37
SMR	*F*_0_/*F* = 0.41 *C*_q_ + 1.54	0.98	1–10	1.60
STZ	*F*_0_/*F* = 0.31 *C*_q_ + 1.61	0.97	1–10	2.07
SIA	*F*_0_/*F* = 0.30 *C*_q_ + 1.27	0.99	1–10	2.15

**Table 3:** Determination of four sulfonamides in water and fish muscle by the UNCP@MIFP-based fluorescent analysis method and HPLC (*n* = 3).

Target		Spiked Concentration (ng/mL or ng/g)	UNCP@MIFP-Based Fluorescent Analysis Method			HPLC			*p* ^b^
			Mean (ng/mL or ng/g)	Recovery (%)	RSD ^a^ (%)	Mean (ng/mL or ng/g)	Recovery (%)	RSD (%)	
Water	SMR	2	1.99	99.67	1.04	1.96	99.00	2.12	0.98
		4	3.98	99.58	1.51	3.93	98.75	1.35	0.31
		8	7.95	99.45	0.71	7.97	100.12	0.52	0.65
	SMZ	2	2.01	100.33	1.60	2.01	101.00	1.04	0.89
		4	4.01	100.17	0.72	3.96	99.25	1.30	0.22
		8	7.95	99.33	1.39	7.96	99.87	0.62	0.89
	STZ	2	1.94	96.83	2.94	1.94	97.50	1.07	0.85
		4	3.92	98.08	1.64	3.94	98.50	0.39	0.63
		8	7.98	99.79	0.81	7.88	99.75	1.06	0.19
	SIA	2	1.94	97.00	2.36	1.91	95.50	1.31	0.43
		4	3.92	98.00	1.17	3.90	99.25	1.80	0.75
		8	7.88	98.50	0.91	7.92	99.50	0.51	0.42
Fish muscle	SMR	2	1.92	96.00	1.56	1.83	96.00	5.98	0.25
		4	3.92	97.92	1.21	3.84	95.50	0.99	0.08
		8	7.92	99.00	0.33	7.89	98.87	1.60	0.68
	SMZ	2	1.93	96.67	1.30	1.99	99.50	2.37	0.13
		4	3.90	97.50	1.93	3.86	96.25	0.93	0.45
		8	7.87	98.37	0.71	7.97	98.87	0.62	0.09
	STZ	2	1.92	96.17	2.46	1.95	96.00	1.36	0.44
		4	3.92	97.92	1.21	3.91	98.25	1.45	0.94
		8	7.90	98.92	1.26	7.90	98.38	0.38	0.73
	SIA	2	1.96	98.17	3.39	1.95	98.50	3.20	0.81
		4	3.91	97.84	0.64	3.93	98.75	0.53	0.46
		8	7.84	98.00	0.63	7.90	98.75	1.59	0.19

^a^ RSD: Relative standard deviation; ^b^ *p* > 0.05 indicates no significant difference.

## Data Availability

The data presented in this work are available in the article.

## Supplementary Materials

The following supporting information can be downloaded at: https://www.mdpi.com/article/10.3390/molecules28083391/s1. Figure S1: Freundlich model for the adsorption of SMZ onto the UCNP@MIFP. Figure S2: Langmuir model for the adsorption of SMZ onto the UCNP@MIFP.

## Sample Availability

Samples of the compounds are not available from the authors.
