# Supplementary material for: A Novel Sulfonamide, Molecularly Imprinted, Upconversion Fluorescence Probe Prepared by Pickering Emulsion Polymerization and Its Adsorption and Optical Sensing Performance
Source: Molecules. 2023 Apr 12;28(8):3391. doi: 10.3390/molecules28083391 (PMC10143443; doi:10.3390/molecules28083391)
Supplement: Supplementary file 1 [file molecules-28-03391-s001.zip › molecules-2324164-supplementary.pdf]

Supplementary Materials

A Novel Sulfonamides Molecularly Imprinted Upconversion Fluorescence Probe Prepared by Pickering Emulsion Polymeri-zation and its Performance of Adsorption and Optical Sensing

Qidi Pan [1], Zhe Gao [1], He Meng [1], Xianghua Guo [2], Meitian Zhang [1] and Yiwei Tang [1,*]

[1] College of Food Science and Technology, Hebei Agricultural University, Baoding 071001, China

[2] Qian'an Agricultural and Rural Bureau, Qian'an 064400, China

***** Correspondence: tangyiwei81@163.com; Tel.: +86 312 7528195

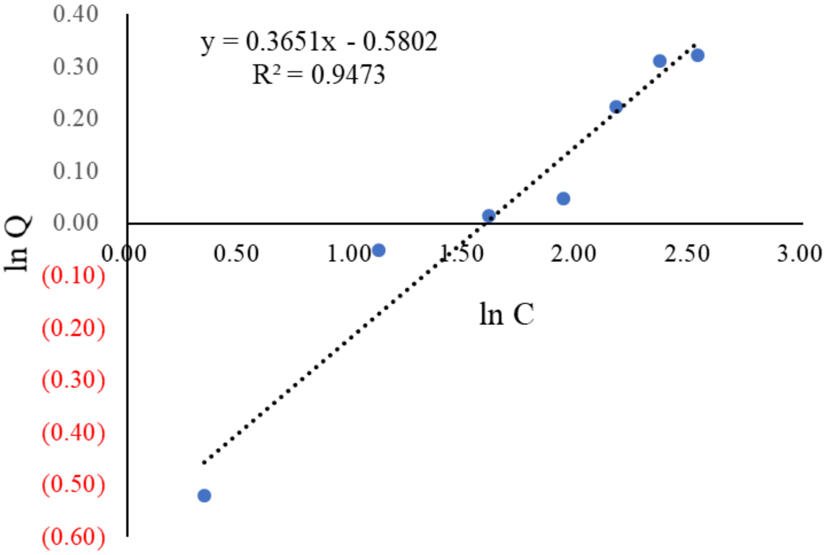

Figure S1 Freundlich model for adsorption of SMZ onto the UCNP@MIFP.

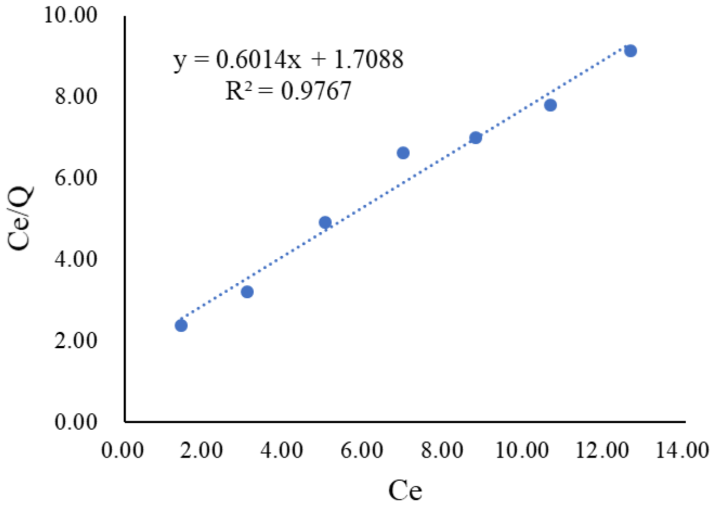

Figure S2 Langmuir model for adsorption of SMZ onto the UCNP@MIFP.
